# High altitude medicine in China in the 21st century: opportunities and challenges

**DOI:** 10.1186/2054-9369-1-17

**Published:** 2014-08-19

**Authors:** Lan Huang

**Affiliations:** Department of Cardiology, Xinqiao Hospital, Third Military Medical University, Chongqing, 400037 China

**Keywords:** High altitude, High altitude disease, Acute mountain sickness, Lake Louise system

## Abstract

China has the largest plateau, Qinghai-Tibet Plateau, where inhabited the most high altitude populations. Moreover, millions of people from plain areas come to the plateau for travel and work purposes and the number of the newcomers has been increasing every year. The hypoxic environment of plateau raised a series of related health issues in the new immigrants, so have created a special medical discipline - High Altitude Medicine. Over the past decades, researches on high altitude medicine have never being ceased in China, and lots of research findings have been reported. Application and practice of these achievements have greatly decreased the mobility and mortality of high-altitude diseases, however, there remained lots of questions to be elucidated. In view of this, the authors were granted a special project from the National Health and Family Planning Commission of China, and conducted a multi-center, prospective, on-scene high altitude medicine study for the acute mountain sickness. Some innovative findings were achieved, and the parameters for diagnosis and application conditions were proposed. Furthermore, the different diagnoses and treatment effects were compared, and a more standardized, reasonable scheme was drawn up. Regarding the unbalanced medical resources in the vast high altitude area, an application system for the public and the army has been established. In the 21st century, innovations in China and novel research approaches have provided great opportunities for the development of high altitude medicine. It is believed that the researchers in China are able to catch the opportunities and address the challenges.

The Qinghai-Tibet Plateau, which is the largest and highest in the world, is located in western China. It is also the home of the largest high-altitude population with a long history. Due to the special and unique environment, its inhabitants have been subjected to physical and psychological challenges. The health problems associated with the exposure to high altitude have long been observed by the local Chinese, mainly the Tibetan and Han populations. Meanwhile, many preventative measures and therapies were developed sporadically. The investigation of high altitude medicine was only initiated after the peaceful liberation of Tibet in 1951. Some of the findings were well accepted by the entire nation and the world.

As part of this investigation, Xinqiao Hospital has played a critical role in high altitude medicine research since the first medical group was organized in this hospital and sent to march to Tibet decades ago. Over the past decades, the impacts of acute exposure to high altitude on the physiological responses, psychological changes, metabolic shifts, pathological changes and disease status changes have been systemically investigated [1]. Many proceedings have been reported in China. For the first time, high altitude pulmonary edema was identified. In addition, the types of acute and chronic high altitude diseases were classified [2]. The clinical symptoms and signs were identified, and the diagnostic criteria were described [3]. The corresponding therapies for these diseases were also described [4]. Meanwhile, the mechanism responsible for hypoxic pulmonary arterial hypertension was illustrated [5, 6]. Overall, much research on the molecular basis and clinical efficacy was well-established after decades of exploration [7, 8]. As a consequence, these achievements have led to decreased incidence and severity of high altitude-associated diseases. The work of other groups has also placed focus on high altitude medicine, which has benefited clinical and basic research in China [9]. Undeniably, China has become a beacon in the field of high altitude research throughout the world.

Currently, the progress in high altitude medicine has facilitated the move of plains residents to high altitude plateaus. As a good example, the Tibet railway was successfully constructed with few health consequences; this was made possible by the contributions of Chinese high altitude medial service providers with their cutting-edge expertise [10–12].

Although such progress has significantly ameliorated the problems associated with rapidly ascending to high altitudes from the plains, minimizing the adversity associated with high altitude diseases remains a problem, especially when large groups are required to ascend to high altitude and perform efficiently in cases such as humanitarian rescue missions after an earthquake. Currently, the international Lake Louise criteria for diagnosing acute high altitude disease differ from China’s criteria [13–15]. The majority of the Chinese experience with high altitude medicine has not been well recognized by the international community. This confusion has resulted in a chaotic state associated with the diagnosis and treatment of high altitude diseases to some extent. Due to the lack of unified guidelines, clinicians vary greatly in their approaches to treating severe acute high altitude diseases. All these phenomena reflect the underdevelopment of high altitude medicine, which cannot meet the demands of the great number of residents in this vast area.

Over the past decades, as economic development has progressed, the population ascending to the high altitude plateau has increased markedly, and human activities and boundaries have gradually been extended. The unbalanced high altitude medical resource configuration did not adapt to the changing situation, which required the further development of high altitude medicine. Meanwhile, several factors have provided unprecedented opportunities for high altitude medicine, including improvements in innovation in China, evidence-based medicine, and translational medicine [16]. In this context, we were granted a special project from the Health Ministry (currently named the National Health and Family Planning Commission of China), and we performed a multi-center, prospective, on-scene high altitude medicine study with many aims, including the establishment of acute high altitude disease alert systems, transformation of our experience-based medicine into evidence-based clinical trials to standardize the identification of high altitude diseases and the application of well-developed techniques to the prevention and treatment of various high altitude diseases [17–19].

In our studies, we discovered some innovative findings regarding some of our objectives, suggesting parameters for diagnosis and application conditions. Furthermore, we compared different diagnoses and treatments and proposed a more standardized, reasonable scheme. Regarding the unbalanced medical resources associated with this vast high altitude area, we established an application system for the public and the army. Many challenges lie ahead and should be faced and dealt with to strengthen high altitude medicine research in China.

The diagnostic criteria differ between China and Western countries. We compared the Chinese criteria and the Lake Louise Consensus, which is widely used in the West. The same groups of participants exposed to rapid high altitude ascension were individually evaluated by these two systems. The results showed that the participants who were diagnosed as having acute high altitude sickness according to the Lake Louise criteria also met the diagnostic criteria of China. However, not all high altitude sickness patients diagnosed by the Chinese criteria were considered to have acute high altitude sickness according to the Lake Louise criteria [18]. Thus, the Lake Louise Consensus appears to have relatively lower sensitivity compared with the Chinese criteria. From another perspective, more false positives might be produced by the Chinese diagnostic criteria compared with the Lake Louise criteria. The conflict between these two criteria creates a dilemma for clinicians in China. The Lake Louise Consensus is well accepted by the international community, which might confuse the interpretation of reports on high altitude sickness from China [14]. Furthermore, the presence of two different criteria increases the heterogeneity among clinical studies and increases the difficulty associated with performing large, multi-site acute high altitude sickness clinical trials. These issues eliminate most Chinese studies, and the paucity of the data produced by Chinese researchers also causes the high altitude sickness results to be less representative, considering the weight of China’s high altitude population and area.

In addition, evidence-based medical studies are limited by a non-linear complex model. Some other affecting factors include the following: geographical area, terrain, geomorphology, climate, weather, sub-environment, participant variables, high altitude exposure categories, duration, acclimatization-adaption types and pre-medication [13]. On-site research also differs from research utilizing hypobaric chambers or other laboratory simulation conditions. All these variables should be considered to minimize any biases and to facilitate the acquisition of more convincing and objective conclusions. It remains unclear how these variables can be reconciled under the principles of evidence-based medicine in real-world studies. Therefore, maintaining the efficacy of the evidence while minimizing the noise signals from other variables remains a priority in evidence-based medicine-guided trials.

In our study, we covered a wide range of ascensions to the high altitude plateau, which were not limited to above 2,500 m sea level. Altitude from 3,700 m to as high as 4,500 m were also investigated, which revealed new effects associated with higher altitude exposure as the body became more hypoxic and the symptoms of acute high altitude sickness developed. Intriguingly, this type of ascension cannot be categorized as acute high altitude sickness, although the hypoxic symptoms are similar. Undoubtedly, this situation leads to an even worse hypoxic status. The precise effects of this type of high altitude exposure remain elusive. Nonetheless, this phenomenon is important because high altitude operations and construction are common.

The high altitude sickness diagnostic criteria used in China differ from the Lake Louise Consensus, which is used in Western countries. Regardless of these distinctions, both sets of criteria adopt complaints as the indices used to diagnose acute high altitude sickness. The caveat is that these evaluation systems are based on subjective complaints rather than objective indices. The identification of severe acute high altitude sickness before the presented clinical symptoms remains a challenge. Considering the on-site conditions, tight cooperation between researchers and clinicians is necessary to apply the techniques used in high altitude medicine research to clinical practice. Current high altitude sickness therapy has already reduced its morbidity and mortality and improved its prognosis. However, the effects greatly differ among different facilities. Evidence-based medicine is an emerging strategy that emphasizes clinical evidence, which serves as a solid and more convincing guide for clinical practice and leads to better prognoses and fewer side effects. Different degrees of recommendations based on the rank of the evidence also promote the widespread use of clinical guidelines and consensus. Introducing this concept into high altitude sickness might provide a new platform to further its research. Guided by evidence-based medicine and combined with newly developed clinical techniques, research on high altitude sickness might advance further. With respect to China’s transitional and empirical methods, some evidence-based advancements should be expected in the near future. Importantly, the cooperation among the sanitary administration, research groups and clinical facilities is critical for the success of these developments. Together, future successful clinical investigations on high altitude sickness require most, if not all, of the following conditions: patient-benefitting moral principles, evidence-based medical guidelines, ease of implementation in the plateau region, and cooperation among different groups.

In this century, it can be foreseen that the population ascending to the plateau will increase and that the extent of human activity will be extended remarkably. Hopefully, research on high altitude medicine in China will blossom based on previous achievements. The introduction of evidence-based medicine and translational medicine might accelerate the development of high altitude medicine in China. Additionally, high altitude medicine fellows in China might contribute more to the world.
